# Wheat CBL-interacting protein kinase 23 positively regulates drought stress and ABA responses

**DOI:** 10.1186/s12870-018-1306-5

**Published:** 2018-05-25

**Authors:** Xiao-Yu Cui, Yong-Tao Du, Jin-dong Fu, Tai-Fei Yu, Chang-Tao Wang, Ming Chen, Jun Chen, You-Zhi Ma, Zhao-Shi Xu

**Affiliations:** 1 Institute of Crop Science, Chinese Academy of Agricultural Sciences (CAAS)/National Key Facility for Crop Gene Resources and Genetic Improvement, Key Laboratory of Biology and Genetic Improvement of Triticeae Crops, Ministry of Agriculture, Beijing, 100081 China; 2 Beijing Advanced Innovation Center for Food Nutrition and Human Health/Beijing Key Lab of Plant Resource Research and Development, Beijing Technology and Business University, Beijing, 100048 China

**Keywords:** ABA, CIPK, CBL, Drought tolerance, Induced mechanism, Wheat

## Abstract

**Background:**

The calcineurin B-like protein (CBL)-interacting protein kinase (CIPK) signaling pathway responds to various abiotic stresses in plants.

**Results:**

Wheat *CIPK23,* isolated from wheat drought transcriptome data set, was induced by multiple abiotic stresses, including drought, salt, and abscisic acid (ABA). Compared with wild-type plants, *TaCIPK23*-overexpression wheat and *Arabidopsis* showed an higher survival rate under drought conditions with enhanced germination rate, developed root system, increased accumulation of osmolytes, and reduced water loss rate. Over-expression of *TaCIPK23* rendered transgenic plants ABA sensitivity, as evidenced by delayed seed germination and the induction of stomatal closure. Consistent with the ABA-sensitive phenotype, the expression level of drought- and ABA-responsive genes were increased under drought conditions in the transgenic plants. In addition, using yeast two-hybrid system, pull-down and bimolecular fluorescence complementation (BiFc) assays, TaCIPK23 was found to interact with TaCBL1 on the plasma membrane.

**Conclusions:**

These results suggest that *TaCIPK23* plays important roles in ABA and drought stress responses, and mediates crosstalk between the ABA signaling pathway and drought stress responses in wheat.

**Electronic supplementary material:**

The online version of this article (10.1186/s12870-018-1306-5) contains supplementary material, which is available to authorized users.

## Background

Plants have developed a broad range of defense strategies and a complex network of signal transduction pathways [1, 2]. Calcium (Ca^2+^) serves as a ubiquitous secondary messenger that is involved in multiple physiological and developmental processes in plants [3, 4]. Unfavorable environmental conditions, such as drought, salt, extreme temperatures, and pathogen infection are all known to trigger discreet spatial and temporal changes in the concentration of [Ca^2+^]_cyt_ in plant cells, leading to specific cellular responses [5]. Ca^2+^ sensor proteins, including Ca^2+^-dependent protein kinases (CDPKs), calmodulins (CAMs), and calcineurin B-like proteins (CBLs), decode these specific signatures and interact with targeted proteins to relay signals [6–8]. Plants have evolved complex Ca^2+^-decoding mechanisms. The CBL-CIPK network is an example of a significantly diverged Ca^2+^-decoding system in plants [9]. CBLs show significant similarity with both the regulatory β subunit of calcineurin (CNB) and the neuronal calcium sensors (NCS) of animals [10]. CBLs harbor four elongation factor (EF) hand motifs as the structural basis for Ca^2+^ binding. These EF hands specifically target a group of CDPKs designated as the CBL-interacting protein kinases (CIPKs) [9]. CIPKs consist of an N-terminal kinase catalytic domain and a C-terminal regulatory domain. The N-terminal kinase catalytic domain is related to sucrose non-fermenting kinase (SNF1) and AMP-activated protein kinase (AMPK) [10, 11]. The C-terminal regulatory domain contains a conserved NAF (Asn-Ala-Phe)/FISL (Phe-Ile-Ser-Leu) motif, consisting of 24 unique amino acid residues, that is essential and sufficient for interaction of CIPK proteins with CBLs [6, 12]. The protein phosphatase interaction (PPI) domain, containing 37 unique amino acid residues, is conserved in *Arabidopsis* protein kinase S (PKS) and in the DNA damage repair and replication block checkpoint kinase, Chk1, from various organisms including humans [13, 14]. The PPI motif, adjacent to the NAF/FISL motif, is necessary for interaction with abscisic acid-insensitive (ABI) protein phosphatases [14].

Comparative genomic analysis of CBL-CIPK genes in plants provides details about the functions, complexity, and conservation of the CBL/CIPK family and the evolution of the CBL-CIPK signaling network [15]. To date, a total of 10 CBLs and 26 CIPKs in *Arabidopsis* and 10 CBLs and 31 CIPKs in rice have been identified [16–18]. Although the specificity of the interactions of most of the CBLs and CIPKs has been confirmed using yeast two-hybrid assay experiments in *Arabidopsis*, the function of CBLs and CIPKs remains elusive [19, 20]. CBL4 interacts with CIPK24 to form specific complexes that function in activating plasma membrane-localized Na^+^/H^+^ antiporters and vacuolar H^+^-ATPases to promote salt tolerance [11]. CIPK21 is involved in the regulation of osmotic stress response in *Arabidopsis* through interaction with the vacuolar Ca^2+^ sensors CBL2 and CBL3 under salt stress conditions [21]. CBL1 and/or CBL9 interact with CIPK23, and control activation of the inward K^+^ channel AKT1, thereby regulating K^+^ uptake under low-K^+^ conditions [22]. The CBL-CIPK signaling pathway plays important roles in plant responses to environmental stresses [23]. To date, investigations of the CBL-CIPK network have mainly concentrated on how ion channels are involved in the influx or efflux of various ions. However, how the CBL-CIPK network participates in drought stress responses in plants have not been extensively reported [22, 24].

Bread wheat (*Triticum avestivum* L.) is one of the global staple crops and is mainly grown in arid and semi-arid regions. Serious water scarcity can cause dramatic yield losses in wheat production systems. Although extensive studies have been made that elucidate the role of the CBL-CIPK signaling pathway in *Arabidopsis*, wheat CIPKs remain poorly investigated; likely owing to the polyploid nature of the bread wheat genome and absence of a complete genome sequence [24, 25]. In the present study, a drought-responsive gene, *TaCIPK23*, was isolated based on a wheat drought de novo transcriptome sequencing experiment [26]. Over-expression of *TaCIPK23* conferred drought tolerance in transgenic wheat and *Arabidopsis*. Further, the *TaCIPK23* transgenic lines were more sensitive to ABA than the wild-type plants. *TaCIPK23* enhanced the expression of a group of drought- and ABA-responsive genes under drought stress conditions. These results reveal a positive role for *TaCIPK23* in conferring drought tolerance and regulating ABA signaling in plants.

## Results

### Identification of drought-responsive CIPK genes in wheat

Previously, the wheat genome was predicted to contain 71 TaCIPK genes [27]. In order to isolate drought-inducible CIPK genes in wheat, we analyzed the expression pattern of TaCIPK genes in wheat drought transcriptome database (http://www.ncbi.nlm.nih.gov/sra) [26]. A total of 21 CIPKs were found to responsive to drought stress at the transcriptional level (Table 1). Among the drought-inducible TaCIPKs, *TaCIPK23* (Genbank No. KD502068) had the most pronounced induction of expression. To explore relationships among these drought-responsive TaCIPKs and previously-reported plant CIPKs, a phylogenetic tree of drought-responsive TaCIPKs and their orthologs from rice, soybean, rapeseed, *Aegilops tauschii*, *Triticum urartu*, maize, sorghum and *Arabidopsis* was constructed. The tree was divided into 7 subgroups. The 21 drought-responsive wheat CIPKs were distributed across 6 subgroups (I, III, IV, V, VI, and VIII; not present in subgroups II,). *TaCIPK23* was included in subgroup I (Additional file 1: Figure S1).

**Table 1:** Wheat CIPKs responsive to drought stress

Gene	Gene ID	CK	Drought	Fold change	Up/Down	*P*-value
TaCIPK2-D	Unigene19129_All	20.8224	61.194	1.5553	Up	4.72E-239
TaCIPK4-B	CL16864.Contig2_All	8.4632	0.7856	−3.4293	Down	5.97E-45
TaCIPK5-A	Unigene2976_All	1.8326	4.2486	1.2131	Up	8.71E-05
TaCIPK10-D	Unigene16768_All	63.5323	6.9792	−3.1864	Down	0
TaCIPK14-A	CL11984.Contig1_All	63.795	9.5433	−2.7409	Down	0
TaCIPK16-B	CL3500.Contig2_All	1.4303	10.6707	2.8993	Up	2.03E-85
TaCIPK16-D	CL3500.Contig1_All	1.2472	7.7425	2.6341	Up	2.03E-55
TaCIPK17-A	CL3945.Contig6_All	3.7199	7.7386	1.0568	Up	7.44E-05
TaCIPK17-B	CL3945.Contig5_All	2.4916	10.8935	2.1283	Up	2.14E-08
TaCIPK17-D	CL3945.Contig3_All	5.4597	19.2913	1.8211	Up	2.15E-32
TaCIPK19-A	CL6163.Contig1_All	3.5214	0.845	−2.0591	Down	2.16E-06
TaCIPK19-B	CL10082.Contig2_All	4.1148	26.2689	2.6745	Up	2.37E-167
TaCIPK21-B	CL13954.Contig1_All	6.1266	28.492	2.2174	Up	4.03E-169
TaCIPK21-D	CL13954.Contig3_All	0.2096	2.1735	3.3743	Up	3.97E-07
TaCIPK23-A	CL5365.Contig4_All	0.9841	23.129	4.5548	Up	3.01E-04
TaCIPK23-B	CL5365.Contig1_All	1.892	8.3534	2.1425	Up	2.97E-47
TaCIPK23-D	CL5365.Contig2_All	0.4497	2.2338	2.3125	Up	4.80E-14
TaCIPK24-D	CL7296.Contig3_All	6.8562	16.3986	1.2581	Up	3.57E-50
TaCIPK26-D	CL6292.Contig1_All	0.9064	2.5742	1.5059	Up	3.60E-12
TaCIPK28-A	Unigene7442_All	1.3571	5.1916	1.9357	Up	1.40E-07
TaCIPK29-B	CL10071.Contig2_All	2.8721	47.0435	4.0338	Up	0

### *TaCIPK23* is induced by multiple abiotic stresses

**Fig. 1:**
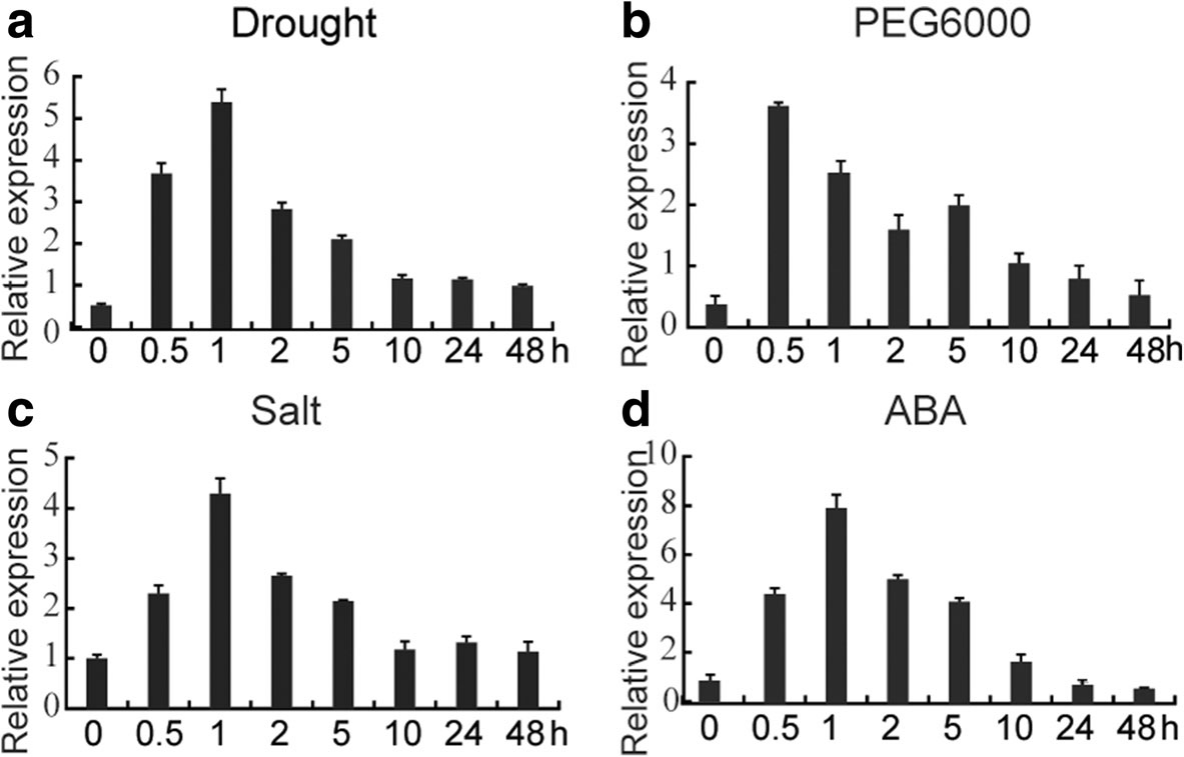
Expression patterns of *TaCIPK23* under various abiotic stresses and subcellular localization of TaCIPK23 in wheat protoplasts. Expression profiles of *TaCIPK23* after treatment with **a** Drought, **b** PEG6000, **c** NaCl, and **d** ABA for 0, 0.5, 1, 2, 5, 10, 24, and 48 h. Transcript levels were quantified by qRT-PCR assays. Expression of *Actin* was analyzed as a loading control. Ten wheat seedlings were combine as a single sample. Each data point is the mean (±SE) of three experiments

To investigate if *TaCIPK23* expression is responsive to diverse stress factors, we conducted quantitative real-time reverse transcription (qRT)-PCR experiments. The expression of *TaCIPK23* was remarkably induced by drought, reaching a peak at 1 h (~ 5-fold) (Fig. 1a). Expression of *TaCIPK23* increased after treatment with 10% PEG6000 and reached a peak (~ 3.5-fold) at 0.5 h (Fig. 1b). Similarly, the expression of *TaCIPK23* was enhanced by salt, reaching peak values (~ 4-fold) at 1 h after treatment (Fig. 1c). *TaCIPK23* expression was also strongly induced by exogenous ABA, reaching its highest level at 1 h (~ 8-fold) (Fig. 1d). The results suggest that *TaCIPK23* is responsive to various abiotic stresses and might function at the intersection of different signaling pathways.

### *TaCIPK23* confers drought resistance in *Arabidopsis* and wheat

**Fig. 2:**
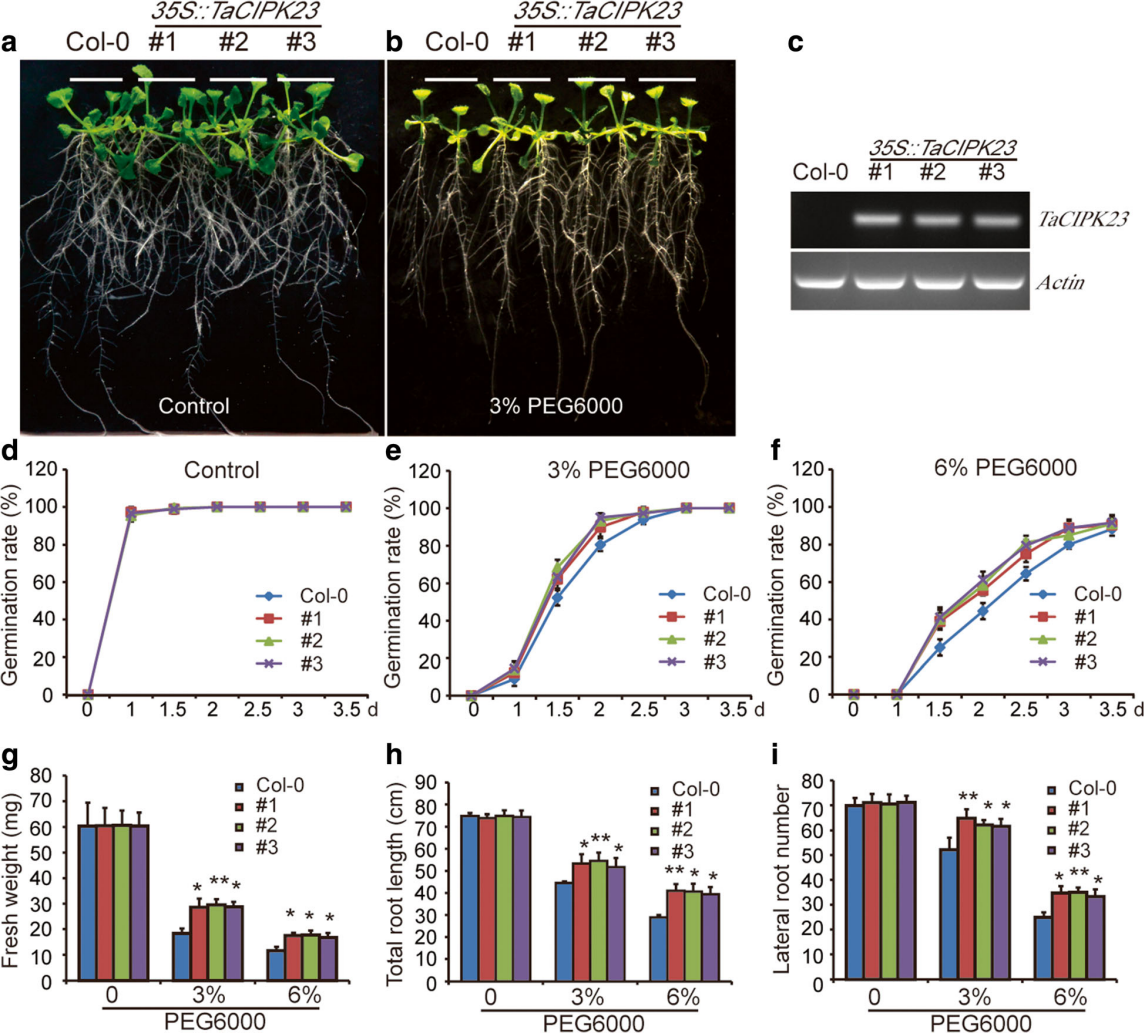
Over-expression of *TaCIPK23* in *Arabidopsis* increased seeds germination rate and root growth under drought treatment. **a**-**b** Five-day-old seedlings grown on 1/2 MS were transferred to 1/2 MS medium containing various concentrations of PEG6000 (0 and 3%). **c** RT-PCR analysis of *TaCIPK23* gene expression from *TaCIPK23*-overexpression and wild-type (Col-0) plants. Expression of *Actin* was analyzed as a loading control. **d**-**f** Time-course of germination percentages of transgenic lines and wild-type seeds in response to PEG6000 treatment. Each data point is the mean (±SE) of three experiments (each with 100 seeds for each line). **g** Measurements of fresh weight. **h** Measurements of total root length. **i** Measurement of lateral roots number. Each data point is the mean (±SE) of three experiments (30 seedlings per experiment). Significant differences from the WT are denoted by two asterisks corresponding to *P* < 0.01 by Student’s t-tests

**Fig. 3:**
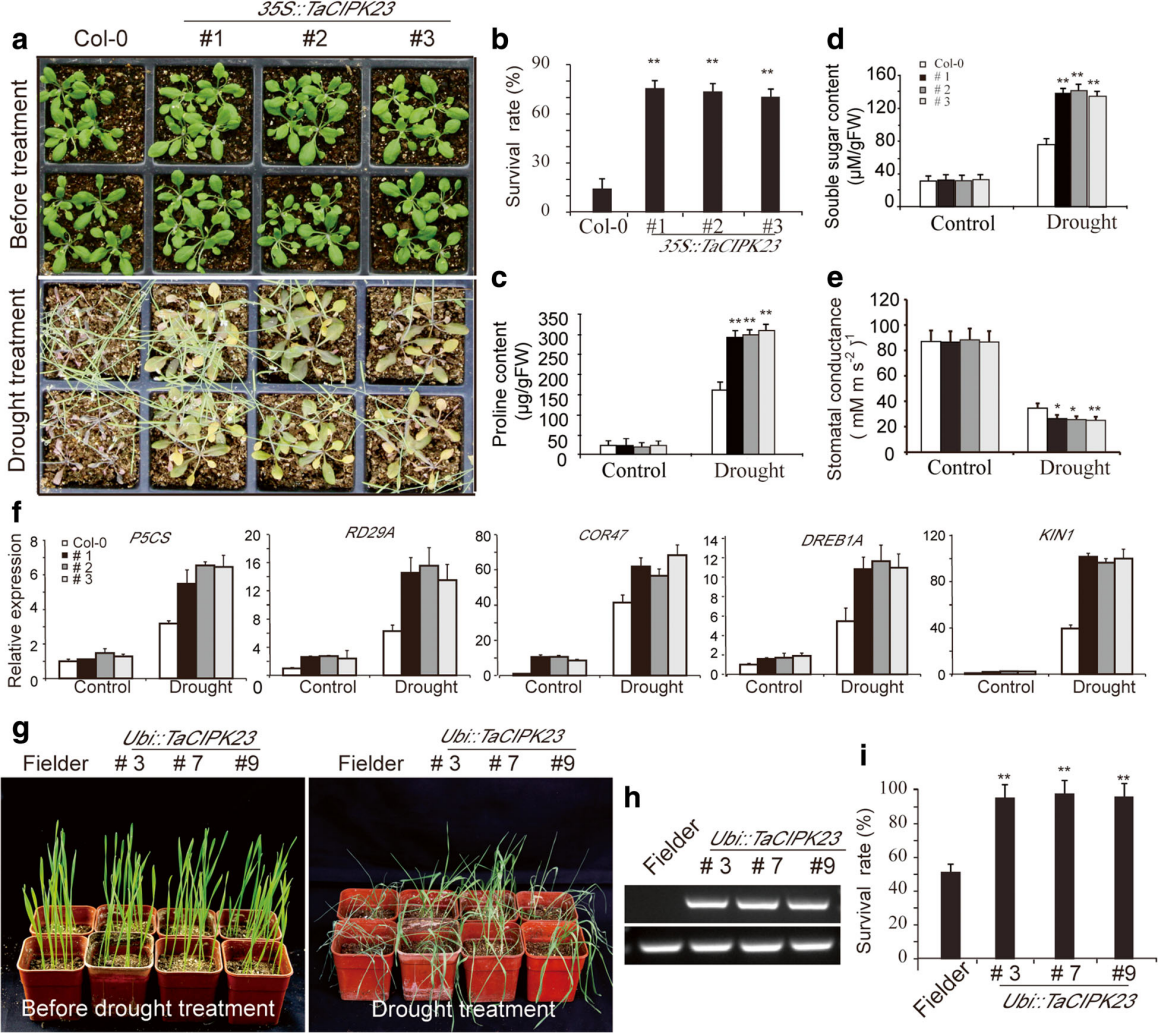
Over-expression of *TaCIPK23* enhanced drought tolerance in transgenic *Arabidopsis* and wheat. **a** Drought tolerance phenotypes of *TaCIPK23*-overexpression lines under water-deficit conditions. 7-day-old *TaCIPK23*-overexpression and wild-type plants were grown for 2 weeks in pots under normal conditions. The plants were subject to another 2-week drought treatment. **b** Survival rate of the water-stressed plants was monitored 5 days after rewatering. **c** Pro content measurement. **d** Souble sugar content measurement. **e** Measurement of stomatal conductance. Each data point is the mean (±SE) of three experiments (10 seedlings per experiment). Significant differences from the WT are denoted by two asterisks corresponding to *P* < 0.01 by Student’s t-tests. **f** The expression level of drought-responsive genes was altered in *TaCIPK23-* overexpressing plants under drought conditions. Two-week-old *TaCIPK23-*overexpressing and wild-type seedlings with 6% PEG6000 treatment were used for RNA isolation. Transcript level was quantified by qRT-PCR assays. Expression of *Actin* was analyzed as a control. Each data point is the mean (±SE) of three experiments (10 seedlings per experiment) Data are means (±SE) of three independent experiments. **g** Drought tolerance phenotypes of *TaCIPK23*-overexpression wheat seedlings under drought conditions. 10-day-old *TaCIPK23*-overexpression and wild-type (Fielder) wheat seedlings were without irrigation for 16 days. **h** RT-PCR analysis of *TaCIPK23* gene expression from *Ubi-TaCIPK23* transgenic and control plants. Expression of *Actin* was analyzed as a loading control. **i** Survival rate of the water-stressed wheat seedlings was monitored 5 days after rewatering

To estimate the capacity of the *TaCIPK23*-overexpression lines to withstand drought, PEG6000 was used to simulate drought stress. Germination percentages were based on radicle emergence after PEG6000 treatment. In the absence of PEG6000, both *TaCIPK23*-overexpression lines and wild-type materials had similar germination (Fig. 2d). However, in the presence of PEG6000, the germination of *TaCIPK23*-overexpression seeds was inhibited less dramatically than that of wild-type seeds (Fig. 2e, f). The *TaCIPK23*-overexpression lines and wild-type seedlings were grown on medium supplemented with PEG6000. As the concentration of PEG6000 (0, 3, and 6%) increased, the growth of wild-type plants was significantly impaired relative to the growth of *TaCIPK23*-overexpression lines (Fig. 2b). Compared with the wild-type plants, the *TaCIPK23*-overexpression lines displayed significantly greater fresh weights, longer root lengths, and a larger number of lateral roots under PEG6000-induced drought stress conditions (Fig. 2g–i). Furthermore, after 3-week-old transgenic and the wild-type seedlings were deprived of water for 2-weeks, the survival rate of *TaCIPK23*-overexpression lines is significantly higher than that of wild-type plants (Fig. 3a, b).

In addition, 10-day-old transgenic and the control wheat seedlings were deprived of water for 16 days. Contrast to the exhibited severe wilting symptoms of wild-type plants, most of the *TaCIPK23*-overexpression lines remained green and wilting slightly (Fig. 3g). Survival rate was monitored 5 days after rewatering. The survival rate of *TaCIPK23* transgenic wheat is significantly higher than that of wild-type plants (Fig. 3i).

To investigate the potential physiological mechanism for the improved drought tolerance of *TaCIPK23*-overexpression lines, the proline and soluble sugar content in wild-type and *TaCIPK23*-overexpression plants were measured under both normal growth and drought conditions. Under normal growth conditions, the proline and soluble sugar content of *TaCIPK23*-overexpression lines and wild-type were similar (Fig. 3c, d). Proline accumulation in the transgenic lines was significantly higher than in wild-type plants under drought conditions (Fig. 3c). *TaCIPK23*-overexpression plants also accumulated higher soluble sugar content than wild-type plants (Fig. 3d). Furthermore, stomatal conductance in the transgenic lines was significantly lower than in wild-type plants under drought conditions (Fig. 3e). These results indicated that the *TaCIPK23*-overexpression lines have improved drought tolerance, likely resulting from accelerated root growth, increased accumulation of osmoprotectants and reduced water loss rate.

To investigate the possible molecular mechanisms of *TaCIPK23* in drought responses, the expression of several drought- and ABA-responsive markers was investigated, including *ABI1* [28], *P5CS* [29, 30], *RD29A* [31], *RD29B* [31], *DREB1A* [32], *COR47* [33], *KIN1* [33], and *ZAT12* [34]. A 2-fold change in expression was arbitrarily considered to be an induction of expression. qRT-PCR analyses revealed that there were no significant differences in the transcript levels of *P5CS, RD29A* and *DREB1A* between *TaCIPK23*-overexpression and wild-type plants under normal conditions. However, under drought stress conditions, the expression of these genes was significantly enhanced in *TaCIPK23*-overexpression lines as compared with the wild-type plants. Compared with the wild-type plants, under normal and drought conditions, the expression of *COR47,* and *KIN1* were was much stronger in the *TaCIPK23*-overexpression lines (Fig. 3f). *ABI1*, *ZAT12,* and *RD29B* were not significantly differentially expressed in the *TaCIPK23*-overexpression lines in either normal or drought conditions (Additional file 1: Figure S2).

### Increased ABA sensitivity in *TaCIPK23* transgenic *Arabidopsis*

**Fig. 4:**
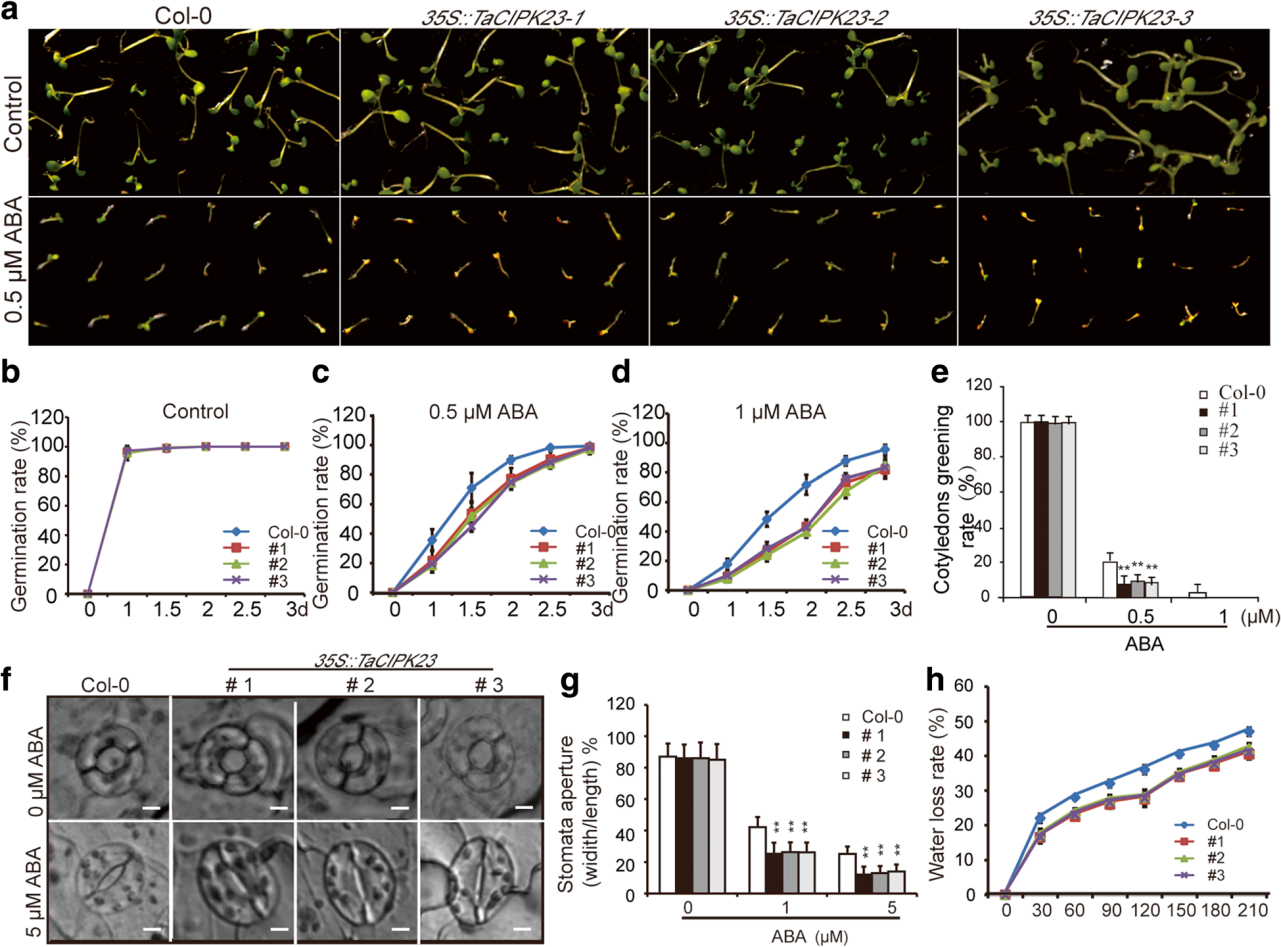
Over-expression of *TaCIPK23* in *Arabidopsis* increased ABA sensitivity. **a** *TaCIPK23*-overexpression and wild-type seeds were germinated on 1/2 MS medium supplemented with different concentrations ABA (0 and 0.5 μM). **b**-**d** Time-course of germination percentages of *TaCIPK23*-overexpression lines and wild-type seeds in response to ABA treatment. **e** Quantitative evaluation of leaf opening and greening rates. Each data point is the mean (±SE) of three experiments (each with 35 seeds for each line). **f** ABA-mediated stomatal closure. 3-week-old rosette leaves of *TaCIPK23*-overexpression lines and wild-type plants grown under continuous light were immersed in stomatal opening solution for 2.5 h and in ABA solution (0, and 5 μM) for an additional 2.5 h. Bars = 10 μm. **g** Measurement of stomatal apertures. **h** Measurement of leaf water loss. Each data point is the mean (±SE) of three experiments (30 stomata per experiment). Significant differences from the WT are denoted by one or two asterisks corresponding to *P* < 0.05 and P < 0.01, respectively, by Student’s t-tests. Significant differences from the WT are denoted by one or two asterisks corresponding to *P* < 0.05 and P < 0.01, respectively, by Student’s t-tests

To examine the role of *TaCIPK23* in plant ABA responses, the seeds from the transgenic lines and wild-type plants were germinated in the presence or absence of ABA (0, 0.5, or 1 μM) and germination percentages (both radicle emergence and cotyledon greening) were calculated from observation data. Germination percentages were based on radicle emergence after ABA treatment. Photographs were taken 5 days after germination. In the absence of ABA, both *TaCIPK23*-overexpression lines and wild-type materials had similar germination (Fig. 4b). However, in the presence of ABA, the seeds of lines *TaCIPK23*-overexpression lines germinated much more slowly than did wild-type seeds (Fig. 4c, d). Differences between *TaCIPK23*-overexpression lines and wild-type seedlings were even more apparent in cotyledon greening (Fig. 4e). These results indicated that germinating *TaCIPK23*-overexpression seeds were more sensitive to ABA than wild-type seeds.

Based on the ABA sensitivity in the germination assay, we tested whether ABA-sensitive stomatal movement in adult plants was altered in the transgenic lines. 3-week-old mature leaves were treated with different concentrations of ABA for 2.5 h, and the length and width of stomata were measured. Stomatal movement profiles were measured as the ratio of width to length [22, 35]. In the absence of ABA treatment, *TaCIPK23*-overexpression and wild-type plants showed comparable average stomatal apertures (Fig. 4f, g). However, after treatment with increasing concentrations of ABA (0, 1, and 5 μM) for 2.5 h, the average stomatal apertures were reduced more dramatically in the *TaCIPK23*-overexpression plants than in wild-type plants (Fig. 4f, g). The water loss rates of detached leaves of the *TaCIPK23*-overexpression lines leaves were lower than those of detached wild-type leaves (Fig. 4h). Plants lose water mainly through stomata. These results suggest that over-expression of *TaCIPK23* can improve ABA sensitivity of plants and can regulate stomatal movement to reduce water loss rate.

### TaCIPK23 interacts with TaCBL1 in vivo and in vitro

**Fig. 5:**
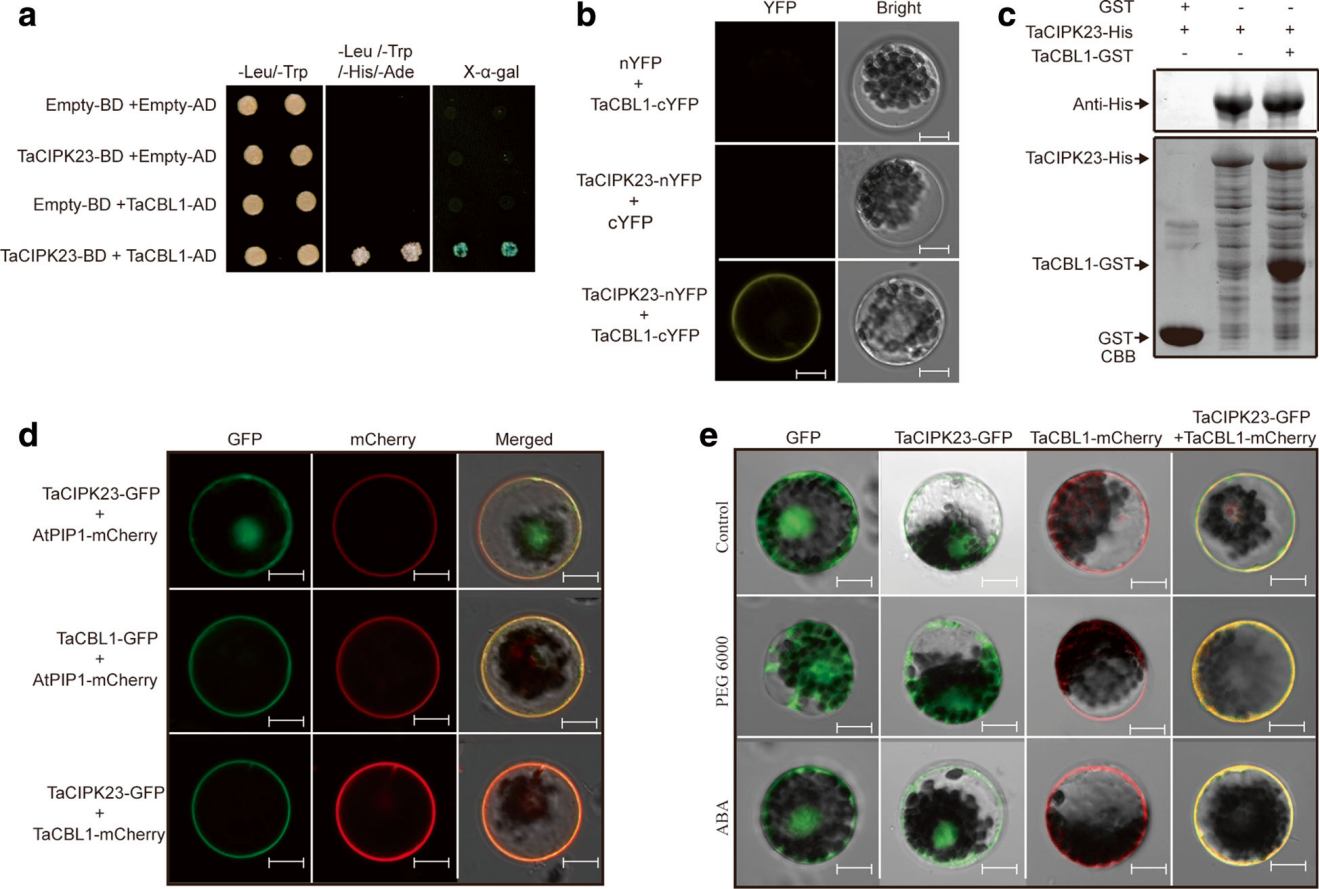
TaCIPK23 interacts with TaCBL1 on the plasma membrane. **a** Yeast two-hybrid analysis of TaCIPK23 interaction with TaCBL1. **b** BiFC assay of the interaction of TaCIPK23 with TaCBL1. Fluorescence and bright field images of wheat protoplast co-transfected with constructs encoding fusion proteins TaCIPK23-YFP^N^ and TaCBL1-YFP^C^. Bar = 12 μm. **c** In vitro pull-down assays were performed by incubating His-TaCIPK23 with GST-TaCBL1 or GST. GST-TaCBL1 was able to pull down His-TaCIPK23 fusion proteins (upper middle panel), but not GST (upper right panel) as detected by Western blot analysis with His antibodies. Coomassie Brilliant Blue (CBB) staining was used to visualize His-TaCIPK23 fusion proteins, GST and the GST-TaCBL1 fusion proteins used in each assay. **d** Localization of TaCIPK23 and TaCBL1 proteins in wheat protoplasts. Images were observed under a laser scanning confocal microscope. Bar = 12 μm. AtPIP1-mCherry protein was used as membrane localization marker. **e** Volume change of wheat protoplast transfected with GFP alone, *TaCBL1*-mCherry alone, *TaCIPK23*-GFP alone, and *TaCBL1*-mCherry/*TaCIPK23*-GFP constructs, respectively, with PEG6000 and ABA treatments. Images were observed under a laser scanning confocal microscope. Bar = 12 μm

To explore the cellular mechanism(s) through which TaCIPK23 participates in drought stress responses, TaCIPK23 was used as bait protein to screen a wheat cDNA library in yeast two-hybrid assays. One interacting candidate, named TaCBL1, was obtained in this experiment (Fig. 5a).

Pull-down experiment was carried out to corroborate the interaction of TaCIPK23 and TaCBL1 in vitro. His-TaCIPK23 and TaCBL1 fused to GST (glutathione-S-transferase) were expressed in *E. coli* (*BL21*). Purified TaCBL1-GST and GST protein were immobilized to glutathione beads, respectively. His-TaCIPK23 protein was incubated with the beads in a pull-down assay. In the analysis, the His-TaCIPK23 protein co-purified with TaCBL1-GST (Fig. 5c; top right panel) but not with GST (Fig. 5c; top left panel), indicating a specific interaction between TaCIPK23 and TaCBL1.

To further corroborate the interaction between TaCIPK23 and TaCBL1 in wheat cells, the *TaCIPK23*-YFP^N^ (N-terminal fragment of yellow fluorescent protein) and *TaCBL1*-YFP^C^ (C-terminal fragment of yellow fluorescent protein) recombination vectors were transformed into wheat protoplasts; fluorescence signals were mainly observed on the plasma membrane. In contrast, no positive signals were observed when *TaCIPK23*-YFP^N^ and YFP^C^ or YFP^N^ and *TaCBL1*-YFP^C^ constructs were co-transformed into wheat protoplasts (Fig. 5b). In addition, subcellular localization assays show that TaCIPK23 was localized to cytoplasm, nucleus, and plasma membrane and TaCBL1 is a membrane-localized protein (Fig. 5d). The AtPIP2*-*mCherry fusion protein was used as plasma membrane marker [36]. When *TaCIPK23*-GFP and *TaCBL1*-mCherry co-expressed in wheat protoplasts, TaCBL1 attached TaCIPK23 to the membrane (Fig. 5d).

To investigate the significance of the direct interaction between TaCIPK23 and TaCBL1, we test volume change of wheat protoplast transfected with GFP alone, *TaCBL1*-mCherry alone, *TaCIPK23*-GFP alone, and *TaCBL1*-mCherry/*TaCIPK23*-GFP constructs, respectively, with PEG6000 and ABA treatments. As is shown in Fig. 5e, the interaction between TaCBL1 and TaCIPK23 was not affected by PEG6000 or ABA treatments. The protoplast volume was similar under normal and ABA conditions. After PEG6000 treatment, protoplasts transfected with GFP constructs was severely damaged, and the volume of these protoplasts was significantly smaller than those protoplasts transfected with *TaCBL1*-mCherry alone, *TaCIPK23*-GFP alone, and both *TaCBL1*-mCherry and *TaCIPK23*-GFP constructs. Compared with protoplasts transfected with *TaCBL1*-mCherry and *TaCIPK23*-GFP, respectively, the lower injury of volume was found in protoplasts co-transfected with *TaCBL1*-mCherry and *TaCIPK23*-GFP constructs. These results suggest that TaCIPK23 maybe interact with TaCBL1 to form a CBL/CIPK complex on the plasma membrane.

## Discussion

Many studies have suggested that the CBL-CIPK signaling pathway plays important roles in abiotic stress responses, including drought stress [37, 38]. Little is known about the diverse functions of wheat CIPK genes in drought response. Understanding the mechanism of wheat CIPKs mediated drought stress response has a great practical benefit for the development of stress-tolerant wheat. In the present study, a CIPK gene, named *TaCIPK23*, was isolated from wheat drought transcriptome databases (Table 1). Biochemical and transgenic studies further supported *TaCIPK23* contributed to plant ABA signaling and drought tolerance.

Transgenic *Arabidopsis* was wildly selected to investigation wheat genes function [39–41]. Although the amino acid sequence of plant CIPKs is highly conserved, their functions are distinctive. Wild-type *Arabidopsis* is selected for transformation to investigate the function of *TaCIPK23* in abiotic stress responses, but not the mutant. In this study, *TaCIPK23*-overexpression plants has a more developed roots system (Fig. 2h, i) and accumulated significantly higher amounts of proline and soluble sugars than did wild-type plants under drought conditions (Fig. 3c, d), a result consistent with the higher survival rate of the *TaCIPK23*-overexpression lines in response to drought stress (Fig. 3b). Root length and lateral root number are phenotype traits for assessing adaptability to environmental stresses [42, 43]. A deep taproot system associated with a moderate number of lateral roots enables plants to absorb enough water and minerals for sustaining the viability of plants [43]. Over-expression of constitutively activated mutant *CaCIPK6* in transgenic tobacco enhanced root growth, which in turn improved its drought and salt tolerance [20]. Under water-deficit conditions, plants accumulate compatible osmolytes such as proline and soluble sugars to protect their subcellular structures from damage [44, 45]. Proline and soluble sugars function in lowering the cellular osmotic potential and restoring intracellular solute concentrations, which prevent water loss from cells [46, 47]. Previous studies reported that the accumulation of Pro and soluble sugars in *OsCIPK03-* and *OsCIPK12*-overexpression plants was significantly higher than that in wild-type plants under abiotic stress conditions, which contributed to the improved tolerance of *OsCIPK03-* and *OsCIPK12-*overexpressing plants to cold and drought stresses, respectively [47]. These results indicated that over-expression of *TaCIPK23* in plant exhibited developed root system and increased the content of osmoprotectants that conferred drought tolerance in transgenic plants. Furthermore, this result was consequently confirmed in wheat (Fig. 3i). To further investigate the role of *TaCIPK23* in abiotic stress responses, we also attempt to knock-down the gene in wheat via RNA interference, but the transcript abundance of *TaCIPK23* was little effect due to the complicated wheat genome. In the future, it is necessary to generate CRISPR-mediated mutants knocking out the three TaCIPK23 homologs in wheat.

ABA plays critical roles in regulating root growth, seed germination, stomatal movement, vegetative growth, and stress responses [7, 48, 49]. Under drought conditions, an elevated level of ABA induces stomatal closure to reduce water loss [46]. ABA-induced stomatal closure represents a major mechanism for plant adaptation to drought [22, 47]. Previous studies reported that *cipk3* loss-of-function mutants were hypersensitive to ABA [7]. *BnCIPK6M* over-expression in transgenic *Arabidopsis* caused hypersensitivity to ABA, whereas silencing of its homologous gene *AtCIPK6* conferred plant ABA insensitive growth phenotypes [50]. In this study, the expression of *TaCIPK23* was induced by exogenous ABA (Fig. 1d). Detailed phenotypic analyses revealed that the *TaCIPK23*-overexpression lines were hypersensitive to ABA with delayed seed germination and small stomatal aperture after exogenous ABA treatment (Fig. 4). Furthermore, the water loss rate of the *TaCIPK23*-overexpression lines was lower than that of wild-type plants (Figs. 3e and 4h). Plants lose water mainly through stomata. The lower stomatal conductance in *TaCIPK23*-overexpression lines causes the reduced water loss rate under drought stress conditions, which contributes the drought tolerance of *TaCIPK23*-overexpression plants. *TaCIPK23* positively regulated several drought- and ABA-responsive genes expression under drought conditions (Fig. 3f). These results indicate that *TaCIPK23* positively modulates plant drought tolerance through ABA-dependent and -independent pathways.

Rapid responses to diverse growth conditions are crucial for plants survival and flourish [51]. Stress perception and signal amplification are involved in stress responses and adaptation [51]. Previous studies indicated that CBL proteins interact with, and are phosphorylated by specific functional interacting CIPKs [51, 52]. Interaction with CBLs is essential for the activation of CIPKs in vivo [53]. Phosphorylation of CBLs by their functional interacting CIPKs enhances complex stability [52]. In vitro kinase activity assays showed that the substrate phosphorylation activity of CIPK24 was negligible in the absence of CBL4, but the CBL4/CIPK24 complex had a basal level of activity for substrate phosphorylation even in the absence of Ca^2+^ [54]. Interaction of SOS2 and SCaBP8, enhanced by SOS2 phosphorylation of SCaBP8 and not requiring Ca^2+^, stabilizes the SCaBP8-SOS2 interaction, which in turn activates plasma membrane Na^+^/H^+^ exchange to improve salt tolerance [55]. Phosphorylation of SCaBP1 by SOS2-LIKE PROTEIN KINASE5 (PKS5) activates their interaction and negatively regulates the activity of AHA2 [56]. In this study, as is illustrated in Fig. 5, TaCIPK23 interacted with TaCBL1 on the plasma membrane. Compared with protoplasts transfected with *TaCIPK23*-GFP vector alone, the lower injury of cellular structure was found in protoplasts co-transfected with *TaCBL1*-mCherry and *TaCIPK23*-GFP constructs. The interaction of TaCIPK23 and TaCBL1 may be able to form a CBL/CIPK complex and enhance the activity of TaCIPK23, contributing to drought stress tolerance.

## Methods

### Plant materials and stress treatment

*Arabidopsis* ecotypes Col-0 was used in this study. Seeds were germinated on 1/2 MS medium (Duchefa) with 2% sucrose and were subsequently transferred to soil. The plants were grown in a greenhouse at 22 °C under long-day conditions (16 h light/8 h dark photoperiod) at a light intensity of around 100 μΜ.m^− 2^ s^− 2^. To generate *TaBZR2D*-overexpressing plants, the coding region of *TaBZR2D* was introduced into the plant transformation vector pBI121 under the control of the *CaMV* 35S promoter. The constructs were confirmed by sequencing and then transformed into wild-type plants (Col-0) by the vacuum infiltration method [57]. Seeds of wild-type and *TaCIPK23*-overpression (independent transgenic lines 1, 2, and 3) plants were sterilized with 30% bleach. After 3 days of stratification at 4 °C, the plates were transferred to a growth chamber. For the germination assay, the sterilized seeds of wild-type and *TaCIPK23*-overpression plants were sown on 1/2 MS growth medium with various concentrations of PEG6000 (0, 3%, or 6%, Merck, USA) or ABA (0, 0.5, or 1.0 μM, Sigma-Aldrich, USA). For the root growth assay, wild-type and transgenic *Arabidopsis* seeds were germinated on MS agar medium for 7 days, followed by transfer to MS growth medium containing various concentrations PEG6000 (0, 3%, or 6%, Merck, USA). Photographs were taken after 7 days of growth and root lengths were evaluated using Epson Expression 11000XL root system scanning analyzer (Epson, Japan). At least 20 seedlings were measured for each genotype. To test drought tolerance at later developmental stages, 14-day-old seedlings were withheld from watering for 14 days, by which time the plants growth were severely affected, and survival rates were calculated for each group of plants. Three independent measurements of 30 seedlings were averaged.

Wheat seedlings (*T. aestivum* L. cultivar Xiaobaimai) were grown in 1/2 Hoagland liquid medium at 22 °C under long-day conditions (16 h light/8 h dark photoperiod) at a light intensity of around 100 μΜ.m^− 2^ s^− 2^ for 2 weeks. For the drought treatment, seedlings were transferred onto filter paper, and dried at 25 °C under 60% humidity conditions. For PEG6000, salt, and ABA treatments, seedling roots were immersed in half-strength Hoagland solution containing 10% PEG6000, 200 mM NaCl, or 100 μM ABA and sampled at 0, 0.5, 1, 2, 5, 10, 24, and 48 h. Harvested seedlings were immediately frozen in liquid nitrogen and stored at − 80 °C prior to RNA extraction. Wheat cultivar fielder was employed as the receptor material to generate the transgenic plants. The ORF of *TaCIPK23* was introduced into the pWMB110 plant transformation vector. The construct was confirmed by sequencing and then transformed into wild-type plants by *Agrobacterium*-mediated wheat transformation system. For the drought tolerance assay, 10-day-old wheat seedlings were deprived of water for 16 d.

### Sequence analysis

The wheat drought de novo transcriptome data was available in the Sequence Read Achive (SRA) accession number SRP071191 [26]. The full-length cDNA of *TaCIPK23* was obtained using gene-specific primers. The PCR products were cloned into the pEASY-T1 vector (TransGene, China) and sequenced. All CIPK sequences in the rice, soybean, rapeseed, *Aegilops tauschii*, *Triticum urartu*, maize, sorghum and *Arabidopsis* genome were identified by using BLASTP, TBLASTN, and the “motif” algorithms to retrieve GenBank database (http://www.ncbi.nlm.nih.gov) [58]. Sequences were aligned with ClustalW using the MEGA5.1 program [59]. The alignment was then used to create a phylogenetic tree with the MEGA5.1 program based on a neighbor-joining method; the confidence level of monophyletic groups was estimated using a bootstrap analysis of 10,00 replicates [59]. The complete amino acid sequence of TaCIPK23 was analyzed with the protein structure prediction tool available at http://www.sbg.bio.ic.ac.uk/phyre2.

### Plasmid construction for localization analysis

The ORF of *TaCIPK23* and *TaCBL1* was cloned into the 16318hGFP vector, respectively and fused with the GFP reporter gene under the control of the cauliflower mosaic virus (CaMV) 35S promoter. The recombinant plasmids were transformed into wheat mesophyll protoplasts with a PEG-mediated method. Expression of fusion proteins was monitored after 12 h of incubation in darkness, and images were captured under a laser scanning confocal microscope (Zeiss LSM700, Germany) [42]. The expressed AtPIP2*-*mCherry fusion protein was used as plasma membrane marker [36].

### Yeast two-hybrid screening

*TaCIPK23* was cloned into the pGBKT7 plasmid. *TaCBL1* was cloned into the pGADT7 plasmid. Yeast strain AH109 expressing pGADT7-*TaCBL1* (prey) was transformed with pGBKT7-*TaCIPK23* (baits). Transformed yeast cells were selected on synthetic complete medium lacking SD/ -Trp/ -Leu following the lithium acetate method (according to TRANFOR protocol). Interaction was determined on synthetic complete medium SD/−Leu/−Trp/-His/−Ade (Sigma-Aldrich, USA) supplemented with 5-Bromo-4-chloro-3-indoxyl-α-D-galactopyranoside (X-α-Gal) (Sigma-Aldrich, USA), at 30 °C for 3 d. Empty prey or bait vectors were transformed with *TaCIPK23* or *TaCBL1* plasmids as negative controls. All bait proteins were tested for self-activation; none were found to activate the reporter genes *LacZ* [21].

### Protein purification and pull-down assays

*TaCIPK23* was inserted into the prokaryotic expression vector pcold TF DNA (TaKaRa, Japan). *TaCBL1* was inserted into the pGEX-4 T-1 plasmid. His-TaCIPK23 and GST-TaCBL1 were expressed in *E. coli* and purified by standard procedures using, respectively, Ni and glutathione agarose beads (GE Healthcare, USA). Briefly, 100 ml of *BL21* cells grown overnight and expressing the desired constructs were transferred into 500 ml of LB and grown at 37 °C for 3 h. Isopropyl-β-d-thiogalactopyranoside (IPTG; 1 mM) was then added to the media and incubated overnight at 16 °C to induce protein expression. The bacterial cells were sonicated in phosphate-buffered saline (PBS) with 1% Triton X-100 and centrifuged at 10, 000 g for 10 min to remove insoluble cell debris. The supernatant was incubated with PBS pre-equilibrated with Ni or lutathione agarose beads and rotated at 4 °C for 2 h. After washing five times with PBS, His-tagged proteins was eluted using 5 mM glutathione and 125 mM imidazole.

For pull-down assays, His-TaCIPK23 was incubated with GST-TaCBL1 or GST in binding buffer [25 mM HEPES (pH 7.6), 12.5 mM MgCl_2_, 150 mM KCl, 0.1% NP-40, and 20% glycerol] buffer for 2 h at 4 °C, respectively. After six washes with buffer [100 mM NaCl, 1 Mm EDTA, 0.5% NP-40, and 20 mM TRIS (pH 8.0)], the proteins were analysed on SDS-polyacrylamide gels followed by immunoblotting using anti-His monoclonal antibodies (NEB, USA) at a 1:1000 dilution. IRDye 800CW anti-mouse IG (H + L) at a 1:15000 dilution (*LI-COR*, USA) was used as the second antibody. The western blots were developed with Odyssey CLx Infrared Imaging Systems (*LI-COR*, USA) [42, 60, 61].

### Bimolecular fluorescence complementation (BiFC) assay

*TaCIPK23* and *TaCBL1* were cloned into the pSPYCE and pSPYNE plasmids, respectively. For the BiFC assays, the *TaCIPK23-*pSPYNE and *TaCBL1-*pSPYCE reconstruction vectors were transformed into common wheat mesophyll protoplasts by a PEG-mediated method. Expression of fusion proteins was monitored after 12 h of incubation in darkness, and images were captured under a laser scanning confocal microscope (Zeiss LSM700, Germany). Yellow fluorescent protein (YFP) fluorescence signals were collected in the 500–570 nm wavelength range. For chloroplast autofluorescence, the wavelength range monitored was 630–700 nm [42, 62].

### qRT-PCR

Total RNA from *Arabidopsis* and wheat seedlings was extracted using an RNAprep plant kit (TIANGEN, China). First-strand cDNA was synthesized using a PrimeScript First-Strand cDNA Synthesis kit (TaKaRa, Japan). The qRT-PCR reactions were performed using an ABI Prism 7500 real-time PCR system (ThermoFisher Scientific, USA) using SYBR Green Master Mix (TIANGEN, China) in a total volume of 25 μl and was performed with three technical replications for each sample. A quantitative analysis was performed using the 2-^ΔΔ^CT method [63].

### Measurements of proline content, soluble sugar content, and stomatal conductance

Seven-day-old *Arabidopsis* seedlings were grown on identical plates filled with a 1:1 mixture of vermiculite and humus. After an additional 3 weeks growth, the seedlings were treated with 10% (*w*/*v*) PEG6000 for 7 additional days. Pro concentration was determined as described [64]; Soluble sugars contents were assayed as described [47]. All the measurements were repeated three times, and the Student’s t-test was used for statistical analysis. For stomatal conductance assay, 3-week-old *Arabidopsis* seedlings were deprive of water for 8 additional days, stomatal conductance was determined as described [65].

### Measurement of water loss rate and stomatal aperture

For the water loss assays, 3-week-old *Arabidopsis* rosette leaves were detached and weighed immediately on a piece of weighing paper and then placed on a laboratory bench and weighed at designated times (0, 30, 60, 120, 180, 210, and 240 min). The percentage loss of fresh weight was calculated on the basis of the initial weight of the detached leaves. The percentage of water loss was calculated as previously described [64]. The experiment was repeated at least three times. Each repetition of the experiment included three replicates for each sampled material.

For the stomatal closure assays, four rosette leaves from 3-week-old plants (grown under 8 h light/16 h dark at 22 °C; 70% relative humidity) were floated in opening buffer (10 mM KCl, 7.5 mM iminodiacetic acid, and 10 mM MES-Tris, pH 6.15) under light [100 μmol/(m^2^. s)] for 4 h, as described previously [35, 51], with some modifications. After the stomata are fully opened, leaves were transferred to ABA-containing buffer (0, 1, and 5μΜ ABA) for 2.5 h for stomatal closing response analysis. The adaxial side of the leaf epidermis was peeled off using tape and observed with a laser scanning confocal microscope (Zeiss LSM700, Germany). Somatal images were photographed with a laser scanning confocal microscope (Zeiss LSM700, Germany), and analyzed using Photoshop CS5 software (Adobe Systems, USA). Stomatal apertures of wild-type and *35S:TaCIPK23* leaves were measured as the ratio of width to length after ABA treatment. Thirty stomata used for analysis were from the central region of the leaves from 3 individual plants.

## Additional file

Additional file 1: **Figure S1.** Phylogenetic tree of CIPK proteins from rice, soybean, rapeseed, *Aegilops tauschii*, *Triticum urartu*, maize, sorghum and *Arabidopsis* . The phylogenetic tree was constructed based on the sequence alignments. The phylogenetic tree of CIPK proteins was constructed with MEGA5.1 program with the neighbor-joining method. The numbers beside the branches represent bootstrap values based on 1000 replications. **Figure S2.** The expression level of drought-responsive genes was altered in *TaCIPK23-* overexpressing plants under drought conditions. Two-week-old *TaCIPK23-*overexpressing and wild-type seedlings with 6% PEG6000 treatment were used for RNA isolation. Transcript level was quantified by qRT-PCR assays. Expression of *Actin* was analyzed as a control. Each data point is the mean (±SE) of three experiments (10 seedlings per experiment). **Table S1.** Primers used for qRT-PCR assays. (PDF 496 kb)

## Abbreviations

[Ca^2+^]cyt: cytosolic Ca^2+^
ABA: Abscisic acid
AMPK: AMP-activated protein kinase
BiFC: Bimolecular fluorescence complementation
Ca^2+^: Calcium
CAMs: Calmodulins
CBL: Calcineurin B-like protein
CIPK: CBL interacting protein kinase
CNB: Regulatory β subunit of calcineurin
NCS: Neuronal calcium sensors
PPI: Protein phosphatase interaction
SNF1: Sucrose non-fermenting kinase
