# Supplementary material for: Wheat CBL-interacting protein kinase 23 positively regulates drought stress and ABA responses
Source: BMC Plant Biol. 2018 May 25;18:93. doi: 10.1186/s12870-018-1306-5 (PMC5970481; doi:10.1186/s12870-018-1306-5)
Supplement: Supplementary file 1 — Figure S1. Phylogenetic tree of CIPK proteins from rice, soybean, rapeseed, Aegilops tauschii, Triticum urartu, maize, sorghum and Arabidopsis . The phylogenetic tree was constructed based on the sequence alignments. The phylogenetic tree of CIPK proteins was constructed with MEGA5.1 program with the neighbor-joining method. The numbers beside the branches represent bootstrap values based on 1000 replications. Figure S2. The expression level of drought-responsive genes was altered in TaCIPK23- overexpressing plants under drought conditions. Two-week-old TaCIPK23-overexpressing and wild-type seedlings with 6% PEG6000 treatment were used for RNA isolation. Transcript level was quantified by qRT-PCR assays. Expression of Actin was analyzed as a control. Each data point is the mean (±SE) of three experiments (10 seedlings per experiment). Table S1. Primers used for qRT-PCR assays. (PDF 496 kb) [file 12870_2018_1306_MOESM1_ESM.pdf]

Wheat CBL-interacting protein kinase 23 positively regulates plant drought stress and ABA responses

*Xiao-Yu Cui [1 †], Yong-Tao Du [1 †], Jin-Dong Fu [1], Tai-Fei Yu [1], Chang-Tao Wang [2], Ming Chen [1], Jun Chen [1] , You-Zhi Ma [1] *, Zhao-Shi Xu [1] **

Figure S1

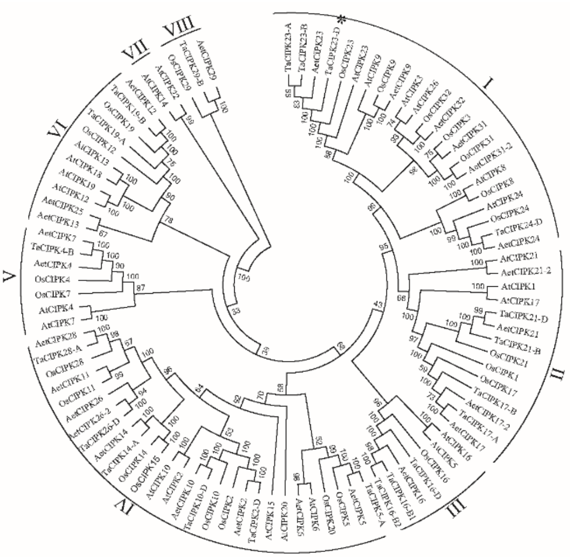

FigureS2

Figure S2

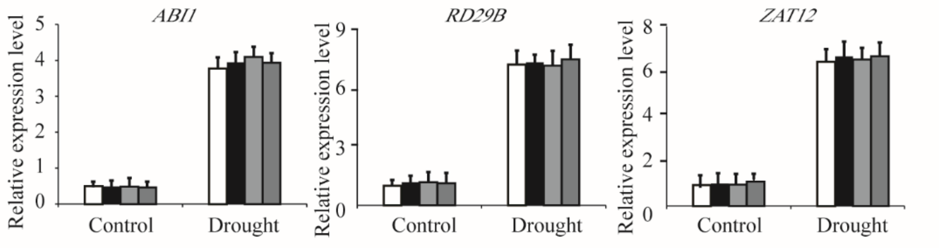

Table S1

Gene	Forward Primer	Reverse Primer
P5CS	GAAGGATTACTTACAACGAGATGGA	CTCTCCTCAAGTCTCAACCAAATAC
NCED3	CAGCTTGTAGCTTTTGGGCTGTA	TAACAGAAACCAGCTGAGCTCGA
RAB18	GCAACTCCACAAGGAAAGTGGT	TGATGACCTGGCAACTTCTCC
RD29A	GGCGTAACAGGTAAACCTAGAG	TCCGATGTAAACGTCGTCC
DREB1A	TCTCTGAACCAGAGTCGTTT	CTTCTTCTCACCGTCTTCAC
COR47	GGAGTACAAGAACAACGTTCCCGA	TGTCGTCGCTGGTGATTCCTCT
KIN1	AACAAGAATGCCTTCCAAGC	CGCATCCGATACACTCTTTCC
ZAT12	GTCACAAGAAGCCTAACAACGA	ACTCAGATCCAAACAAGCCAC
RD22B	ATACCTTCCGACCAGATAGCGG	TACCACCGAGCCAAGAAGTGAC
Arabidopsis actin	AAGCAATGAGCATGAGCAAG	GGAAGACACGACAGGAAACAC
TaCIPK23	ATGGCAGGCTACCTTCCGTT	GATTGAGGCCCTGGGAGGTA
wheat actin	CTCCCTCACAACAACCGC	TACCAGGAACTTCCATACCAAC
